# Multicenter observational study of patients who underwent cardiac surgery and were hospitalized in an intensive care unit (BraSIS 2): study protocol and statistical analysis plan

**DOI:** 10.62675/2965-2774.20250222

**Published:** 2025-02-27

**Authors:** Nair Naiara Barros de Vasconcelos, Renato Carneiro de Freitas Chaves, Carolina de Moraes Pellegrino, Guilherme Martins de Souza, Veronica Neves Fialho Queiroz, Carmen Silvia Valente Barbas, Flávio Takaoka, Ricardo Luiz Cordioli, Sandrigo Mangini, Fabio de Vasconcelos Papa, Hélio Penna Guimarães, Adriano José Pereira, Ary Serpa, Andre Gulinelli, Anna Clara Legal, Caio Vinicius Gouvêa Jaoude, Eduardo Paolinelli, Eric Benedet Lineburger, Erick César de Farias Albuquerque, Evaldo Gomes Ferreira, Fabio Barlem Hohmann, Felipe Galdino, Felipe Souza Lima Vianna, Frederico Toledo Campo Dall’Orto, Lucas Tramujas, Luciano Ribeiro Pereira Silva, Maxim Goncharov, Paulo César Gottardo, Roberto Rabello, Thais Dias Midega, Vinicius Barbosa Galindo, Vinícius Caldeira Quintão, Viviane Cordeiro Veiga, Thiago Domingos Corrêa, João Manoel Silva

**Affiliations:** 1 Hospital Israelita Albert Einstein São Paulo SP Brazil Hospital Israelita Albert Einstein - São Paulo (SP), Brazil.; 2 St. Michael’s Hospital Toronto Canada St. Michael’s Hospital - Toronto, Canada.; 3 Hospital São José Criciúma SC Brazil Hospital São José - Criciúma (SC), Brazil.; 4 Hospital Alberto Urquiza Wanderley João Pessoa PB Brazil Hospital Alberto Urquiza Wanderley - João Pessoa (PB), Brazil.; 5 Irmandade da Santa Casa da Misericórdia de Santos Santos SP Brazil Irmandade da Santa Casa da Misericórdia de Santos - Santos (SP), Brazil.; 6 Hospital Maternidade e Pronto Socorro Santa Lúcia Minas Gerais MG Brazil Hospital Maternidade e Pronto Socorro Santa Lúcia - Minas Gerais (MG), Brazil.; 7 Instituto de Pesquisa HCor-Hospital do Coração São Paulo SP Brazil Instituto de Pesquisa, HCor-Hospital do Coração - São Paulo (SP), Brazil.; 8 Hospital Nossa Senhora das Neves João Pessoa PB Brazil Hospital Nossa Senhora das Neves - João Pessoa (PB), Brazil.; 9 Hospital das Clínicas Faculdade de Medicina Universidade de São Paulo São Paulo SP Brazil Academic Research Organization, Instituto do Coração, Hospital das Clínicas, Faculdade de Medicina, Universidade de São Paulo - São Paulo (SP), Brazil.; 10 A Beneficência Portuguesa de São Paulo São Paulo SP Brazil BP - A Beneficência Portuguesa de São Paulo, São Paulo (SP), Brazil.

**Keywords:** Cardiac surgery, Critical care, Anesthesia, Mortality, Epidemiology

## Abstract

**Background:**

The perioperative management of patients undergoing cardiac surgery is highly complex and involves numerous factors. There is a strong association between cardiac surgery and perioperative complications. The Brazilian Surgical Identification Study (BraSIS 2) aims to assess the incidence of death and early postoperative complications, identify potential risk factors, and examine both the demographic characteristics of patients and the epidemiology of cardiovascular procedures.

**Methods and analysis:**

BraSIS 2 is a multicenter observational study of patients who undergo cardiac surgery and who are admitted to the intensive care unit. The primary objective is to describe the risk factors and incidence of mortality or severe postoperative complications occurring within the first 3 postoperative days of cardiac surgery or until intensive care unit discharge (whichever event occurs first). Severe postoperative complications include acute myocardial infarction, acute respiratory distress syndrome, cardiorespiratory arrest with return of spontaneous circulation, Kidney Disease Improving Global Outcomes stage ≥ 2, a new surgical approach being conducted in an unscheduled event of urgency or emergency, renal replacement therapy, septic shock, severe bleeding, severe hemodynamic instability, stroke, unplanned reintubation, and unplanned use of a circulatory assistance device. The secondary outcomes include the evaluation of patient characteristics and descriptions of the performed surgeries and administered anesthesia. This study will also assess intraoperative and postoperative complications, as well as risk factors associated with postoperative complications and mortality. We expect to recruit 500 patients from at least 10 Brazilian intensive care units. Trial registration: NCT06154473; partial results.

## BACKGROUND

Cardiac surgery represents an important therapeutic intervention for patients with various cardiovascular diseases, although it is accompanied by inherent risks and complexities.^(1)^ Both open-heart surgery and percutaneous procedures have become commonplace in contemporary medical practice.^(1,2)^ Despite advancements in surgical and anesthetic techniques, which have contributed to a decreased overall perioperative mortality rate for cardiac procedures, significant challenges continue to persist.^(3-5)^

Whereas cardiac surgery demonstrates potential in improving quality of life and increasing survival rates among patients with cardiovascular disease, it is not without risks and complications.^(4,5)^ The management of patients during the perioperative period is characterized by high complexity and numerous challenges.^(6)^ Factors such as baseline patient conditions, complexities of surgeries, and postoperative complications contribute to elevated in-hospital and postdischarge mortality rates.^(7-9)^ The clinical outcomes of patients undergoing cardiac surgery are closely associated with institutional expertise, thus underscoring the importance of tailored interventions to mitigate the risks of mortality or severe complications.

An understanding of major preoperative risk factors is crucial for optimizing perioperative management and improving patient outcomes.^(10)^ However, there is a notable scarcity of Brazilian data regarding patients undergoing cardiac surgery, thereby highlighting the need for comprehensive studies to enhance the understanding of cardiac surgery risks in this population.

The Brazilian Surgical Identification Study (BraSIS 2) aims to investigate the incidence of death and early severe postoperative complications, identify potential risk factors, and examine both the demographic characteristics of patients and the epidemiology of cardiovascular procedures.

## METHODS

### Study design and setting

This multicenter prospective observational study was designed according to the guidelines for Good Clinical Practice and the Declaration of Helsinki and was registered at www.clinicaltrials.gov (trial identification number: NCT06154473). The main characteristics of the BraSIS 2 study are summarized in the Synopsis table (Table 1).

**Table 1:** Synopsis (ClinicalTrials.gov registration, as originally submitted)

Data category	Information
Primary registration and identification number	ClinicalTrials.gov – NCT06154473
Date of first registration	2023-07-25
Financial support	None
General and academic contact	RCFC, MD, PhD, MBA Phone: +55 (11) 2151-1500 E-mail: renato.carneiro@einstein.br
Primary sponsor	*Hospital Israelita Albert Einstein*
Public title	Assessment of patients undergoing cardiac surgery and admitted to the intensive care unit (BraSIS-2)
Academic title	Assessment of patients undergoing cardiac surgery and admitted to the intensive care unit: an observational, prospective, multicenter study
Countries involved in recruitment	Brazil
Health conditions/investigated problems	Cardiac surgery, intensive care unit care, postoperative complications
Main inclusion criteria	Adult patients undergoing cardiac surgery requiring postoperative care in the intensive care unit will be included Age: ≥ 18-years-old Sex: both Accepts volunteers: no
Main exclusion criteria	Exclusive palliative care, advance directive expressing desire for limitation of life support, patients previously included in this study, surgery for implantation of cardiac implantable electronic device and moribund patient
Type of study	Observational, prospective and multicenter study
Expected date of first inclusion	September 2023
Sample size	500 patients
Recruitment status	Not initiated (expected for 2023)
Primary outcome	Mortality or serious postoperative complications within the first 3 postoperative days or until ICU discharge (whichever event occurs first)
Secondary outcomes	− Evaluate patient characteristics − Describe the performed surgeries and anesthesia − Assess intraoperative and postoperative complications − Assess risk factors associated with postoperative complications and mortality

### Patient and public involvement

Patients or the public were not involved in the study design.

### Patient eligibility

Consecutive patients who meet the inclusion criteria and none of the exclusion criteria will be enrolled in this study. Patients who undergo cardiac surgery and who are hospitalized in an intensive care unit (ICU) will be consecutively included by each of the participating centers during the study period. The patients will be eligible for inclusion in the study during the ICU stay and until the third postoperative day. After the third day following surgery or after discharge from the ICU, the patients will no longer be eligible for inclusion.

### Sites and recruitment

**Figure 1:**
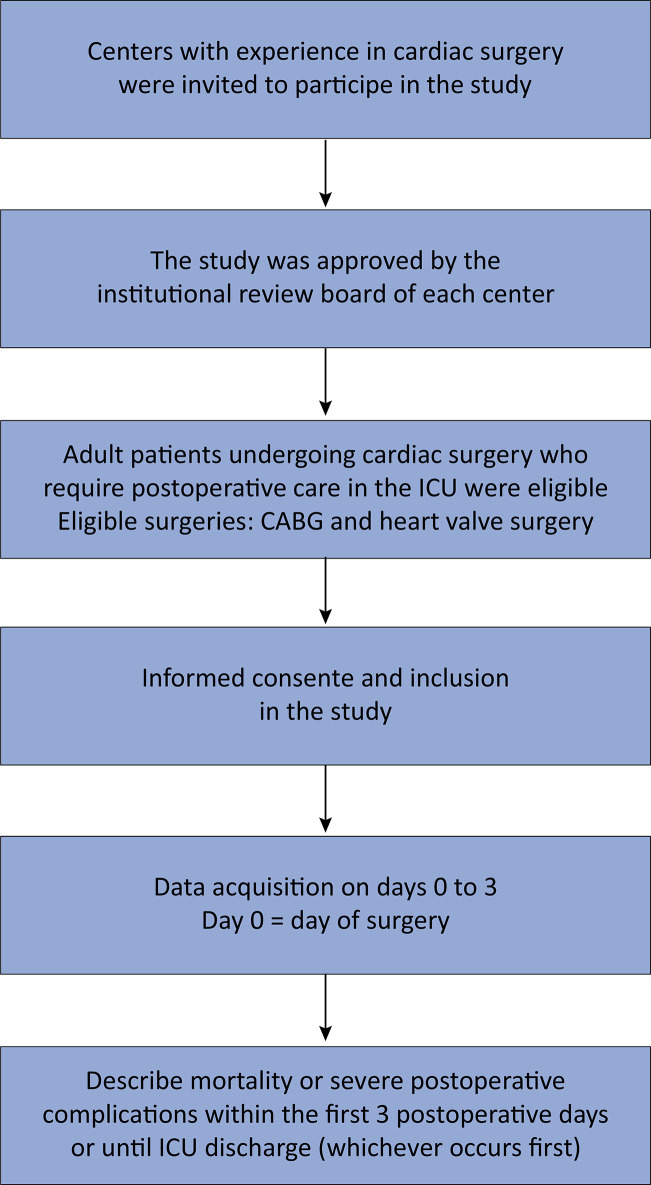
Study flowchart. CABG - coronary artery bypass grafting; ICU - intensive care unit.

Centers with experience in cardiac surgery were invited to participate in this study (Figure 1). At least 10 Brazilian ICUs will be included. The expected recruitment rate is 5 patients/month/site, with a planned study duration estimated at 1.5 years.

### Inclusion and exclusion criteria

Adult patients aged ≥ 18 years who are undergoing cardiac surgery and who require postoperative care in the ICU will be included. Eligible surgeries include coronary artery bypass grafting and heart valve surgery (both open and percutaneous surgeries/procedures). Patients receiving exclusive palliative care before surgery, patients with advance directives expressing a desire for limitation of life support before surgery, patients who were previously included in the BraSIS 2 study, patients undergoing surgery for the implantation of cardiac implantable electronic devices, and patients in a moribund state will be excluded.

### Follow-up

Patients will be followed up until discharge from the ICU or until the third postoperative day (whichever event occurs first) by the health care worker responsible for data collection.

### Objectives

The primary objective of this study is to describe the risk factors and incidence of mortality or severe postoperative complications within the first 3 postoperative days or until ICU discharge (whichever event occurs first). Severe postoperative complications include acute myocardial infarction, acute respiratory distress syndrome (ARDS) with partial pressure of oxygen (PaO_2_)/fraction of inspired oxygen (FiO_2_) ≤ 250 (refractory to rescue maneuvers and ventilatory adjustments that persist for more than 1 hour), cardiorespiratory arrest with the return of spontaneous circulation, Kidney Disease Improving Global Outcomes stage (KDIGO) ≥ 2,^(11)^ a new surgical approach being used in an unscheduled event of urgency or emergency, renal replacement therapy, septic shock, severe bleeding, severe hemodynamic instability, stroke, unplanned reintubation, and unplanned use of a circulatory assistance device. The clinical outcomes are presented in mock table 2.

**Table 2:** Clinical outcomes

	All patients (n =)	Open-heart surgery (n =)	Percutaneous surgery (n =)
**Primary outcomes**			
ICU mortality or severe postoperative complications	n/Total (%)	n/Total (%)	n/Total (%)
ICU mortality	n/Total (%)	n/Total (%)	n/Total (%)
Severe postoperative complications	n/Total (%)	n/Total (%)	n/Total (%)
Acute myocardial infarction	n/Total (%)	n/Total (%)	n/Total (%)
ARDS	n/Total (%)	n/Total (%)	n/Total (%)
Cardiorespiratory arrest with return of spontaneous circulation	n/Total (%)	n/Total (%)	n/Total (%)
KDIGO ≥ 2	n/Total (%)	n/Total (%)	n/Total (%)
New surgical approach in an unscheduled event of urgency or emergency	n/Total (%)	n/Total (%)	n/Total (%)
Renal replacement therapy	n/Total (%)	n/Total (%)	n/Total (%)
Septic shock	n/Total (%)	n/Total (%)	n/Total (%)
Severe bleeding	n/Total (%)	n/Total (%)	n/Total (%)
Severe hemodynamic instability	n/Total (%)	n/Total (%)	n/Total (%)
Stroke	n/Total (%)	n/Total (%)	n/Total (%)
Unplanned reintubation	n/Total (%)	n/Total (%)	n/Total (%)
Unplanned use of a circulatory assistance device	n/Total (%)	n/Total (%)	n/Total (%)
**Secondary outcomes**			
Intraoperative complications	n/Total (%)	n/Total (%)	n/Total (%)
Bronchospasm or difficulties in respiratory support	n/Total (%)	n/Total (%)	n/Total (%)
Extracorporeal circulation output failure	n/Total (%)	n/Total (%)	n/Total (%)
Cardiac arrest with return of spontaneous circulation	n/Total (%)	n/Total (%)	n/Total (%)
Excessive blood transfusion	n/Total (%)	n/Total (%)	n/Total (%)
Length of stay in the ICU	n/Total (%)	n/Total (%)	n/Total (%)
Postoperative complications	n/Total (%)	n/Total (%)	n/Total (%)
Cardiac tamponade	n/Total (%)	n/Total (%)	n/Total (%)
Dysrhythmias	n/Total (%)	n/Total (%)	n/Total (%)
Postoperative nausea and vomiting	n/Total (%)	n/Total (%)	n/Total (%)
Paralytic ileus	n/Total (%)	n/Total (%)	n/Total (%)
Pneumonia	n/Total (%)	n/Total (%)	n/Total (%)
Pneumothorax	n/Total (%)	n/Total (%)	n/Total (%)
Psychomotor agitation	n/Total (%)	n/Total (%)	n/Total (%)
Sustained diarrhea	n/Total (%)	n/Total (%)	n/Total (%)
Unplanned need for oxygen supplementation	n/Total (%)	n/Total (%)	n/Total (%)

ARDS - acute respiratory distress syndrome; KDIGO - Kidney Disease Improving Global Outcomes stage; ICU - intensive care unit.

Acute myocardial infarction is defined by the universal classification of type 5 infarction, which is characterized by an elevation of troponin at 10 times the 99th percentile associated with one of the following changes: the development of new pathological Q waves; newly documented angiographic graft occlusion or new native coronary artery occlusion; or imaging evidence of new losses of viable myocardium or new regional wall motion abnormalities in a pattern that is consistent with an ischemic etiology.^(12)^ Acute respiratory distress syndrome is defined according to the Berlin definition, whereby cases explained by heart failure or volume overload are excluded.^(13)^ Severe bleeding is defined as a decrease of ≥ 2g/dL of hemoglobin or transfusion of 2 units of packed red blood cells without an increase in the hemoglobin value, a decrease in the systolic blood pressure ≥ 10mmHg upon standing, a spontaneous decrease in the systolic blood pressure ≥ 20mmHg, or a heart rate increase ≥ 20 beats per minute.^(14,15)^ Severe hemodynamic instability is defined as norepinephrine ≥ 0.1mcg/kg/min or epinephrine ≥ 0.1mcg/kg/min for more than 2 hours, the independent combination of norepinephrine and epinephrine (regardless of the dose) or an independent combination of norepinephrine and vasopressin.^(16,17)^

The secondary objectives include descriptions of anesthesia and surgical procedures, the incidence of intraoperative complications such as bronchospasms or respiratory support difficulties, failure to separate from cardiopulmonary bypass (at least one failure of the first separation attempt or the need for a circulatory assistance device to exit the operating room), cardiac arrest with the return of spontaneous circulation, excessive blood transfusion (greater than 4 units of packed red blood cells), the incidence of postoperative complications that are not included in the primary outcome such as cardiac tamponade, arrhythmias (including atrial fibrillation, atrial flutter, sustained ventricular tachycardia, supraventricular tachycardia, and ventricular fibrillation), length of stay in the ICU, postoperative nausea and vomiting that is difficult to control, paralytic ileus, patient characteristics, pneumonia, pneumothorax, psychomotor agitation (defined as a Richmond Agitation-Sedation Scale [RASS] score ≥ +2),^(18)^ risk factors associated with mortality, risk factors associated with postoperative complications, sustained diarrhea, and the unplanned need for oxygen supplementation.

### Steps and data collection

Local investigators at each participating center will screen all patients undergoing cardiac surgery. Mortality and postoperative complications will be recorded on Day 0 (from the end of surgery until 11:59 pm) and on postoperative Days 1, 2, and 3 (each day from 00:00 to 23:59). Data collection will continue until the day of ICU discharge or until Day 3 for patients who remain hospitalized in the ICU. The start date for each participating center is flexible and will be determined collaboratively with the study coordinator, following approval by the Institutional Review Board of each participating center. The characteristics of the included patients are presented in table 3, the intraoperative data and complications are presented in table 4, the types of performed surgery are presented in table 5, and factors related to the risk of mortality or severe postoperative complications are presented in table 6.

**Table 3:** Characteristics of the included patients

	All patients (n=)	Open-heart Surgery (n=)	Percutaneous surgery (n=)
Age (years)	Mean ± SD	Mean ± SD	Mean ± SD
ASA score	Mean ± SD	Mean ± SD	Mean ± SD
Male sex	n/Total (%)	n/Total (%)	n/Total (%)
BMI (kg/m^2^)	Mean ± SD	Mean ± SD	Mean ± SD
EUROSCORE	Mean ± SD	Mean ± SD	Mean ± SD
SOFA score	Mean ± SD	Mean ± SD	Mean ± SD
Comorbidities			
Active cancer	n/Total (%)	n/Total (%)	n/Total (%)
Active endocarditis in use of antibiotic therapy at the time of surgery	n/Total (%)	n/Total (%)	n/Total (%)
Acute myocardial infarction less than 90 days prior to study	n/Total (%)	n/Total (%)	n/Total (%)
Anemia	n/Total (%)	n/Total (%)	n/Total (%)
Asthma	n/Total (%)	n/Total (%)	n/Total (%)
Atrial fibrillation or atrial flutter	n/Total (%)	n/Total (%)	n/Total (%)
Chronic renal failure on renal replacement therapy	n/Total (%)	n/Total (%)	n/Total (%)
COPD	n/Total (%)	n/Total (%)	n/Total (%)
Coronary heart disease	n/Total (%)	n/Total (%)	n/Total (%)
Diabetes mellitus	n/Total (%)	n/Total (%)	n/Total (%)
Heart failure via the NYHA functional classification	n/Total (%)	n/Total (%)	n/Total (%)
Class I	n/Total (%)	n/Total (%)	n/Total (%)
Class II	n/Total (%)	n/Total (%)	n/Total (%)
Class III	n/Total (%)	n/Total (%)	n/Total (%)
Class IV	n/Total (%)	n/Total (%)	n/Total (%)
Left ventricular dysfunction	n/Total (%)	n/Total (%)	n/Total (%)
Preserved function (LVEF ≥ 51%)	n/Total (%)	n/Total (%)	n/Total (%)
Intermediate function (LVEF 40 - 50%)	n/Total (%)	n/Total (%)	n/Total (%)
Reduced function (LVEF 30 - 39%)	n/Total (%)	n/Total (%)	n/Total (%)
Very reduced function (LVEF ≤ 29%)	n/Total (%)	n/Total (%)	n/Total (%)
Liver cirrhosis	n/Total (%)	n/Total (%)	n/Total (%)
Obstructive sleep apnea	n/Total (%)	n/Total (%)	n/Total (%)
Previous heart surgery (a stay in another hospital)	n/Total (%)	n/Total (%)	n/Total (%)
Pulmonary hypertension	n/Total (%)	n/Total (%)	n/Total (%)
Rheumatic fever	n/Total (%)	n/Total (%)	n/Total (%)
Systemic arterial @hypertension	n/Total (%)	n/Total (%)	n/Total (%)
Stroke	n/Total (%)	n/Total (%)	n/Total (%)
Unstable angina for less than 90 days	n/Total (%)	n/Total (%)	n/Total (%)
Laboratory tests before surgery			
Hemoglobin (g/dL)	Mean ± SD	Mean ± SD	Mean ± SD
Creatinine (mg/dL)	Mean ± SD	Mean ± SD	Mean ± SD
Ionic calcium (mg/dL)	Mean ± SD	Mean ± SD	Mean ± SD
Glucose (mg/dL)	Mean ± SD	Mean ± SD	Mean ± SD
BNP (pg/mL)	Mean ± SD	Mean ± SD	Mean ± SD

SD - standard deviation; ASA - American Society of Anesthesiology; BMI - body mass index; EUROSCORE - European System for Cardiac Operative Risk Evaluation; SOFA - Sequential Organ Failure Assessment; COPD - chronic obstructive pulmonary disease; NYHA - New York Heart Association; LVEF - left ventricular ejection fraction; BNP - B-type natriuretic peptide.

**Table 4:** Intraoperative data and complications

	All patients (n =)	Open-heart surgery (n =)	Percutaneous surgery (n =)
Anesthesia administered	n/Total (%)	n/Total (%)	n/Total (%)
General anesthesia	n/Total (%)	n/Total (%)	n/Total (%)
Combined anesthesia	n/Total (%)	n/Total (%)	n/Total (%)
Total intravenous drugs	Mean ± SD	Mean ± SD	Mean ± SD
Duration of the surgical procedure, minutes	Mean ± SD	Mean ± SD	Mean ± SD
Type of surgery performed	n/Total (%)	n/Total (%)	n/Total (%)
Urgent/emergency	n/Total (%)	n/Total (%)	n/Total (%)
Elective	n/Total (%)	n/Total (%)	n/Total (%)
Extracorporeal circulation	n/Total (%)	n/Total (%)	n/Total (%)
Transfusion of blood products	n/Total (%)	n/Total (%)	n/Total (%)
Red blood cells	n/Total (%)	n/Total (%)	n/Total (%)
Cryoprecipitate	n/Total (%)	n/Total (%)	n/Total (%)
Fresh frozen plasma	n/Total (%)	n/Total (%)	n/Total (%)
Platelets	Mean ± SD	Mean ± SD	Mean ± SD
Urine output (mL)	Mean ± SD	Mean ± SD	Mean ± SD
Total fluid intake (mL)	Mean ± SD	Mean ± SD	Mean ± SD
Crystalloids (mL)	Mean ± SD	Mean ± SD	Mean ± SD
Colloid (mL)	Mean ± SD	Mean ± SD	Mean ± SD
Complications during anesthesia	n/Total (%)	n/Total (%)	n/Total (%)
Bronchoaspiration	n/Total (%)	n/Total (%)	n/Total (%)
Bronchospasm or difficulties in breathing support	n/Total (%)	n/Total (%)	n/Total (%)
Extracorporeal circulation output failure	n/Total (%)	n/Total (%)	n/Total (%)
Cardiopulmonary arrest with return to spontaneous circulation	n/Total (%)	n/Total (%)	n/Total (%)
Excessive blood transfusion	n/Total (%)	n/Total (%)	n/Total (%)

SD - standard deviation.

**Table 5:** Type of surgery performed

Open surgery	n/Total (%)
Valvuloplasty	n/Total (%)
Aortic valve	n/Total (%)
Pulmonary valve	n/Total (%)
Mitral valve	n/Total (%)
Tricuspid valve	n/Total (%)
Valve replacement	n/Total (%)
Aortic valve	n/Total (%)
Pulmonary valve	n/Total (%)
Mitral valve	n/Total (%)
Tricuspid valve	n/Total (%)
Type of implanted valve	n/Total (%)
Mechanical	n/Total (%)
Biological	n/Total (%)
Revascularization of the myocardium	n/Total (%)
Number of bridges performed	Mean ± SD
Percutaneous surgery	n/Total (%)
TAVI	n/Total (%)
ViV-TAVI	n/Total (%)
Transcatheter treatment of the mitral valve	n/Total (%)
Transcatheter bicaval valves system	n/Total (%)

SD - standard deviation; TAVI - transcatheter aortic valve implantation; ViV-TAVI - valve-in-valve TAVI.

**Table 6:** Factors related to the risk of mortality or severe postoperative complications

	All patients (n =)	Univariate analyses		Multivariate analyses	
		OR (95%CI)	p value	OR (95% CI)	p value
Risk of mortality or severe postoperative complications					
Factors	n/Total (%)	OR (95%CI)	p value	OR (95%CI)	p value
Factors	n/Total (%)	OR (95%CI)	p value	OR (95%CI)	p value
Risk of mortality					
Factors	n/Total (%)	OR (95%CI)	p value	OR (95%CI)	p value
Factors	n/Total (%)	OR (95%CI)	p value	OR (95%CI)	p value
Risk of severe postoperative complications					
Factors	n/Total (%)	OR (95%CI)	p value	OR (95%CI)	p value
Factors	n/Total (%)	OR (95%CI)	p value	OR (95%CI)	p value
Patients undergoing open-heart surgery					
Risk of mortality or severe postoperative complications					
Factors	n/Total (%)	OR (95%CI)	p value	OR (95%CI)	p value
Factors	n/Total (%)	OR (95%CI)	p value	OR (95%CI)	p value
Risk of mortality					
Factors	n/Total (%)	OR (95%CI)	p value	OR (95%CI)	p value
Factors	n/Total (%)	OR (95%CI)	p value	OR (95%CI)	p value
Risk of severe postoperative complications					
Factors	n/Total (%)	OR (95%CI)	P value	OR (95%CI)	p value
Factors	n/Total (%)	OR (95%CI)	P value	OR (95%CI)	p value
Patients undergoing percutaneous surgery					
Risk of mortality or severe postoperative complications					
Factors	n/Total (%)	OR (95%CI)	p value	OR (95%CI)	p value
Factors	n/Total (%)	OR (95%CI)	p value	OR (95%CI)	p value
Risk of mortality					
Factors	n/Total (%)	OR (95%CI)	p value	OR (95%CI)	p value
Factors	n/Total (%)	OR (95%CI)	p value	OR (95%CI)	p value
Risk of severe postoperative complications					
Factors	n/Total (%)	OR (95%CI)	p value	OR (95%CI)	p value
Factors	n/Total (%)	OR (95%CI)	p value	OR (95%CI)	p value

OR - odds ratio; 95%CI - 95% confidence interval.

### Data collection and management

Data will be entered using an electronic case report form in the Research Electronic Data Capture (REDCap®, USA) platform by using the internet, with the platform hosted on a server at the *Hospital Israelita Albert Einstein*, São Paulo, Brazil.^(19,20)^ This system possesses various functionalities, including patient registration, data entry, data validation, data reporting, data quality evaluation, data resolution workflows, audit trials, and data export for statistical analysis.^(19,20)^ Local investigators will directly input data into the system, with comprehensive usage instructions always being available to the investigators. Electronic files will be securely archived on the *Hospital Israelita Albert Einstein* server in a controlled and confidential environment, and they will be safeguarded by using password protection protocols following best practices.

Regular remote data monitoring will promptly identify irregular patterns, inconsistencies, credibility concerns, or anomalies using predefined queries within the system. Missing or outlier data values will be individually reviewed, and follow-up reports will be regularly reviewed by the coordinating center to ensure consistency and completeness. Efforts will be made to complete or rectify data whenever possible.

### Cleaning and locking of the database

The database will be locked once all of the data have been entered, as well as when all discrepancies or missing data have been addressed. If all attempts to resolve the remaining issues are unsuccessful, the database will be considered for locking. Prior to database locking, a thorough review of the data will be conducted. Subsequently, the study database will be locked and prepared for statistical analysis. At this step, access permissions to the database will be revoked for all of the investigators, and the database will be archived.

### Auditing

The BraSIS 2 study is subjected to audit by the Einstein Research Integrity Committee at any time, independent of the Institutional Review Board (IRB) and the research team and following the same procedure as any other study performed at *Hospital Israelita Albert Einstein* (with the study being randomly selected).

### Calculation of sample size

We expect to recruit 500 consecutive patients who are undergoing cardiac surgery. Based on previous findings, the incidence of severe complications in the postoperative period of cardiovascular surgeries within the first 3 postoperative days or until discharge from the ICU is approximately 20%.^(21)^ Therefore, by enrolling 500 patients, we aim to develop a robust regression model with up to 10 independent variables. To increase the representativeness of the findings, the maximum number of patients that a single center could enroll in the study was limited to 100 patients. Additionally, there is no limit on the number of patients who undergo open or percutaneous surgery. Patients will be consecutively enrolled, and this approach aims to capture the typical practices in managing patients undergoing cardiac surgery at the participating centers.

### Statistical analysis plan

Data will be collected as part of routine clinical care. Patient characteristics will be compared and described using appropriate methods. Continuous numerical variables will be evaluated for distribution patterns via histograms and the Kolmogorov-Smirnov test. Qualitative variables will be expressed as proportions, and quantitative variables will be expressed as the means ± standard deviations or as medians and 25^th^ and 75^th^ percentiles (interquartile ranges), when appropriate.

The number of patients who die or exhibit severe postoperative complications will be reported in absolute numbers and percentages. The primary outcome will be assessed via a multiple logistic regression model. A multiple logistic regression model will be employed to identify the risk factors associated with postoperative complications and mortality. Separate models will be used to identify the risk factors that are specific to postoperative complications and those associated with mortality. Additionally, separate models will also be employed to identify the risk factors that are specific to patients undergoing open surgery and patients undergoing percutaneous surgery. Given the hierarchical nature of the data, all of the models will consider the center as a random effect in the intercept. The relevant covariates that are included in the final multivariable model will be identified as those with p < 0.2 in the univariable model, as well as those covariates with clinical relevance and no statistical associations with other relevant variables.

Minimal missing values for exploratory outcomes are anticipated, as the study procedures include the thorough training of site research staff and independent remote and onsite data monitoring conducted by the study coordinator. Multiple imputation will be performed if the percentage of missing data regarding the core variables exceeds 10%, following standard procedures for multiple imputation using chained equations. For a percentage of missing data ≤ 10%, complete case analyses will be conducted.

Exploratory analysis will be conducted to assess the risk factors that are associated with postoperative complications and mortality. This exploratory analysis will be performed using the paired t test (or Wilcoxon signed rank test for nonnormally distributed data) if a time effect is detected, as well as by using Cox regression; moreover, visualization will be performed using the Kaplan-Meier curve.

After data collection from the last enrolled patient is completed, the database will be cleaned and locked, and the analysis plan will be submitted for publication. The hypothesis tests will be two-sided with a significance level of 5%, and no adjustments of p values will be made for multiple comparisons. All of the analyses will be performed using R software version 4.2.0 or using the most updated version (R Foundation for Statistical Computing, Vienna, Austria).

Subgroups of patients undergoing open surgery or percutaneous surgery will be separately analyzed. Prespecified secondary outcomes and subgroup analyses will not be adjusted for multiple comparisons; therefore, they should be interpreted as exploratory factors. We have prespecified the following risk factor analyses to assess their impacts on mortality and postoperative complications: patients with a previous use of a circulatory assistance device compared to those without prior use, patients with prior uses of vasoactive drugs compared to those without preoperative uses, comparison of the types of utilized cardioplegic solutions, comparison of the types of utilized circulatory assistance devices, influence of cumulative fluid balance, influence of hyperglycemia requiring intravenous insulin, influence of laboratory tests collected during the perioperative period, myocardial revascularization with extracorporeal circulation *versus* without extracorporeal circulation, impact of cardiopulmonary bypass time, impact of intraoperative use of etomidate, impact of preoperative anemia, impact of the development of anemia during the perioperative period, and impact of the type of infused solution (crystalloid or colloid). Additionally, we will describe the risk factors that are associated with the development of the following postoperative complications: renal complications, pulmonary complications, cardiovascular complications, neurological complications, gastrointestinal complications, hematological complications, new surgical approaches conducted in an unscheduled event of urgency or emergency, sepsis and septic shock, and severe hemodynamic instability.

### Collected variables

Demographic data and preoperative, intraoperative and postoperative variables will be collected. During the postoperative follow-up period, variables will be collected in the first three postoperative days or until discharge from the ICU (whichever event occurs first). Data will be recorded using printed clinical forms and/or an electronic form that is specifically designed for the study by utilizing the electronic form REDCap®. Access to the system will be individually provided to each study participant, and this access will be secured via login and password procedures. The authors will be responsible for monitoring the data. The variables that are collected from each patient will be described below.

### Randomization

Not applicable.

### Ethical considerations

This study will be performed according to national and international guidelines, adhering to the principles of the Declaration of Helsinki and the Act for Medical Research Involving Humans. This study will be approved by the local Research Ethics Committee (Institutional Review Board, or IRB) of the coordinating study center (*Hospital Israelita Albert Einstein*) (CAAE: 69330823.1.0000.0071), as well as by the local IRB from each center, in compliance with Brazilian legislation. Sites will be required to obtain documentation of proof that the IRB evaluated and approved the study. Any modifications to the protocol that may affect the development, potential benefits, or safety of the study (including changes in the objectives, design, study population, sample size, interventions or relevant management aspects) require protocol amendments. These amendments should be submitted for approval to the IRB of the coordinating center and to all of the IRBs at the participating centers. The need for informed consent is determined by the Institutional Review Board of each participating center.

### Safety

#### Adverse events and interim analyses

Due to the fact that this study is observational and that we do not anticipate inherent risks in its performance, interim analyses are not planned. Adverse events are defined as unwanted events experienced by a patient during the study, regardless of whether they are related to the proposed interventions or not. Although adverse events related to the study are not expected, local researchers, data assistants, and attending physicians are responsible for reporting any such events to the research ethics committee that approved the study.

#### Patient information and informed consent

The researchers will request written consent from the patient or their legal representative if the patient’s clinical conditions do not allow them to directly provide consent. The proposed informed consent form will be evaluated by each research center, and any necessary changes must be approved by the coordinating center of the study before submission to the IRB. If informed consent is not required by the local IRB, a waiver must be obtained.

Either the investigators at each site or the study coordinator will be responsible for obtaining consent and providing all of the relevant information regarding the study to the patient or their legal representative. The patient (or the patient’s legal representative) and the researcher assigned to obtain consent must date and sign two copies of the informed consent form, with one copy being provided to the patient (or their legal representative) and the other copy being filed with the study documents. Researchers must ensure that patients or their legal representatives understand that participating in the study is voluntary and that they may withdraw from the study at any time without affecting the quality and conduct of the subsequent medical treatment.

## Confidentiality of data

The patient and the investigating center will be identified by corresponding numbers on the electronic data collection form to ensure anonymity. The data obtained from medical records must be handled in a confidential manner and stored in cabinets with restricted access by the researchers. Anonymity of all of the data in both provisional and definitive reports will be guaranteed, thereby ensuring that no identifiable information is disclosed. Sites that are involved in the research must securely store all of the data for the duration specified by the study and according to local regulations. After the designated study period has elapsed, data must be securely destroyed to prevent unauthorized access to patient data. However, the team will enact every precaution to ensure data confidentiality throughout the study and beyond.

## Publication and administrative aspects

### Coordinating center

The coordinating center of the study is the *Hospital Israelita Albert Einstein*, São Paulo, Brazil. The responsibilities of the coordinating center include planning and conducting the study, preparing the protocol and clinical forms for data collection, developing the operations manual, managing and controlling the quality of the data, performing the statistical analysis, and preparing the final manuscript.

## Public disclosure and publication policy

The BraSIS 2 group will publish the study findings regardless of the results. The main manuscript will be submitted on behalf of the research group (BraSIS 2).

## Organization

The coordinators will be responsible for recruiting sites and ensuring the proper performance of the study. Local coordinators at each participating center will provide scientific and structural leadership. They will ensure that all of the necessary local ethical and regulatory approvals are obtained before patient enrollment begins. Local coordinators will also train and monitor their respective research teams, thus ensuring the integrity of data collection and the inclusion of data in the electronic medical records.

## References

[1] Serruys PW, Morice MC, Kappetein AP, Colombo A, Holmes DR, Mack MJ, Ståhle E, Feldman TE, van den Brand M, Bass EJ, Van Dyck N, Leadley K, Dawkins KD, Mohr FW, SYNTAX Investigators (2009). Percutaneous coronary intervention versus coronary-artery bypass grafting for severe coronary artery disease. N Engl J Med.

[2] Tsao CW, Aday AW, Almarzooq ZI, Anderson CA, Arora P, Avery CL, Baker-Smith CM, Beaton AZ, Boehme AK, Buxton AE, Commodore-Mensah Y, Elkind MSV, Evenson KR, Eze-Nliam C, Fugar S, Generoso G, Heard DG, Hiremath S, Ho JE, Kalani R, Kazi DS, Ko D, Levine DA, Liu J, Ma J, Magnani JW, Michos ED, Mussolino ME, Navaneethan SD, Parikh NI, Poudel R, Rezk-Hanna M, Roth GA, Shah NS, St-Onge MP, Thacker EL, Virani SS, Voeks JH, Wang NY, Wong ND, Wong SS, Yaffe K, Martin SS, American Heart Association Council on Epidemiology and Prevention Statistics Committee and Stroke Statistics Subcommittee (2023). Heart Disease and Stroke Statistics-2023 Update: A Report From the American Heart Association. Circulation.

[3] Flynn M, Reddy S, Shepherd W, Holmes C, Armstrong D, Lunn C (2004). Fast-tracking revisited: routine cardiac surgical patients need minimal intensive care. Eur J Cardiothorac Surg.

[4] Dieberg G, Smart NA, King N (2016). Minimally invasive cardiac surgery: a systematic review and meta-analysis. Int J Cardiol.

[5] Yusuf S, Zucker D, Peduzzi P, Fisher LD, Takaro T, Kennedy JW (1994). Effect of coronary artery bypass graft surgery on survival: overview of 10-year results from randomised trials by the Coronary Artery Bypass Graft Surgery Trialists Collaboration. Lancet.

[6] Landoni G, Augoustides JG, Guarracino F, Santini F, Ponschab M, Pasero D (2011). Mortality reduction in cardiac anesthesia and intensive care: results of the first International Consensus Conference. Acta Anaesthesiol Scand.

[7] Nashef SA, Roques F, Sharples LD, Nilsson J, Smith C, Goldstone AR (2012). EuroSCORE II. Eur J Cardiothorac Surg.

[8] Edwards FH, Clark RE, Schwartz M (1994). Coronary artery bypass grafting: the Society of Thoracic Surgeons National Database experience. Ann Thorac Surg.

[9] Farooq V, van Klaveren D, Steyerberg EW, Meliga E, Vergouwe Y, Chieffo A (2013). Anatomical and clinical characteristics to guide decision making between coronary artery bypass surgery and percutaneous coronary intervention for individual patients: development and validation of SYNTAX score II. Lancet.

[10] Silva JM, Chaves RC, Corrêa TD, Assunção MS, Katayama HT, Bosso FE (2020). Epidemiology and outcome of high-surgical-risk patients admitted to an intensive care unit in Brazil. Rev Bras Ter Intensiva.

[11] Levin A, Stevens PE, Bilous RW (2013). Kidney disease: improving global outcomes (KDIGO) CKD work group. KDIGO 2012 clinical practice guideline for the evaluation and management of chronic kidney disease. Kidney Int Suppl.

[12] Thygesen K, Alpert JS, Jaffe AS, Chaitman BR, Bax JJ, Morrow DA, White HD, Executive Group on behalf of the Joint European Society of Cardiology (ESC)/American College of Cardiology (ACC)/American Heart Association (AHA)/World Heart Federation (WHF) Task Force for the Universal Definition of Myocardial Infarction (2018). Fourth Universal Definition of Myocardial Infarction (2018). Circulation.

[13] Ranieri VM, Rubenfeld GD, Thompson BT, Ferguson ND, Caldwell E, Fan E, ARDS Definition Task Force (2012). Acute respiratory distress syndrome: the Berlin Definition. JAMA.

[14] Dyke C, Aronson S, Dietrich W, Hofmann A, Karkouti K, Levi M (2014). Universal definition of perioperative bleeding in adult cardiac surgery. J Thorac Cardiovasc Surg.

[15] Rocha LL, Neto AS, Pessoa CM, Almeida MD, Juffermans NP, Crochemore T (2020). Comparison of three transfusion protocols prior to central venous catheterization in patients with cirrhosis: A randomized controlled trial. J Thromb Haemost.

[16] Argenziano M, Chen JM, Choudhri AF, Cullinane S, Garfein E, Weinberg AD (1998). Management of vasodilatory shock after cardiac surgery: identification of predisposing factors and use of a novel pressor agent. J Thorac Cardiovasc Surg.

[17] Buitenwerf E, Boekel MF, van der Velde MI, Voogd MF, Kerstens MN, Wietasch GJ (2019). The haemodynamic instability score: development and internal validation of a new rating method of intra-operative haemodynamic instability. Eur J Anaesthesiol.

[18] Ely EW, Truman B, Shintani A, Thomason JW, Wheeler AP, Gordon S (2003). Monitoring sedation status over time in ICU patients: reliability and validity of the Richmond Agitation-Sedation Scale (RASS). JAMA.

[19] Harris PA, Taylor R, Minor BL, Elliott V, Fernandez M, O'Neal L, McLeod L, Delacqua G, Delacqua F, REDCap Consortium (2019). The REDCap consortium: building an international community of software platform partners. J Biomed Inform.

[20] Van Bulck L, Wampers M, Moons P (2022). Research Electronic Data Capture (REDCap): tackling data collection, management, storage, and privacy challenges. Eur J Cardiovasc Nurs.

[21] Ball L, Costantino F, Pelosi P (2016). Postoperative complications of patients undergoing cardiac surgery. Curr Opin Crit Care.

